# Unlocking the global health potential of dried blood spot cards

**DOI:** 10.7189/jogh.12.03027

**Published:** 2022-07-16

**Authors:** Brianne Bota, Victoria Ward, Monica Lamoureux, Emeril Santander, Robin Ducharme, Steven Hawken, Beth K Potter, Raphael Atito, Bryan Nyamanda, Stephen Munga, Nancy Otieno, Sowmitra Chakraborty, Samir Saha, Jeffrey SA Stringer, Humphrey Mwape, Joan T Price, Hilda Angela Mujuru, Gwendoline Chimhini, Thulani Magwali, Pranesh Chakraborty, Gary L Darmstadt, Kumanan Wilson

**Affiliations:** 1 Clinical Epidemiology Program, Ottawa Hospital Research Institute, Ottawa, Canada; 2 Prematurity Research Center, Department of Pediatrics, Stanford University School of Medicine, Stanford, California, USA; 3 Newborn Screening Ontario, Children’s Hospital of Eastern Ontario, Ottawa, Canada; 4 Kenya Medical Research Institute (KEMRI), Center for Global Health Research, Kisumu, Kenya; 5 Child Health Research Foundation, Mirzapur, Bangladesh; 6 Department of Obstetrics and Gynecology, UNC School of Medicine, Chapel Hill, North Carolina, USA; 7 UNC Global Projects Zambia, Lusaka, Zambia; 8 Department of Paediatrics and Child Health, University of Zimbabwe, Harare, Zimbabwe; 9 Department of Obstetrics and Gynaecology, Faculty of Medicine and Health Sciences, University of Zimbabwe, Harare, Zimbabwe; 10 School of Epidemiology and Public Health, University of Ottawa, Ottawa, Canada; 11 Department of Pediatrics, University of Ottawa, Ottawa, Canada; 12 Department of Medicine, University of Ottawa, Ottawa, Canada; 13 Bruyere Research Institute, Ottawa, Ontario

A key challenge to providing care for newborn infants in low- and middle-income countries (LMICs) is the lack of timely diagnostic testing due to weak local infrastructures such as laboratory capacity and imaging technologies. To address this challenge, low-cost diagnostic testing that does not require extensive laboratory processing or costly storage procedures has been prioritised. One such example is the shipping of newborn dried blood spot (DBS) samples to international centres where screening for metabolic disorders can be conducted.

DBS samples are used as a source of metabolites for newborn screening in many places around the world [1]. These samples contain information on the metabolic “fingerprint” of infants which can be used to identify whether they are at risk for specific inborn errors of metabolism and other genetic conditions. We previously demonstrated that data from DBS-derived metabolites can also be utilised to accurately estimate gestational age [2-6]. Gestational age dating is challenging in LMICs due to the lack of access to prenatal ultrasound and the unreliability of dating based on the last menstrual period [7,8]. Accurate population-level estimations of preterm birth are critical in informing preconception and prenatal care initiatives and prioritisation in funding distribution.

**Figure:**
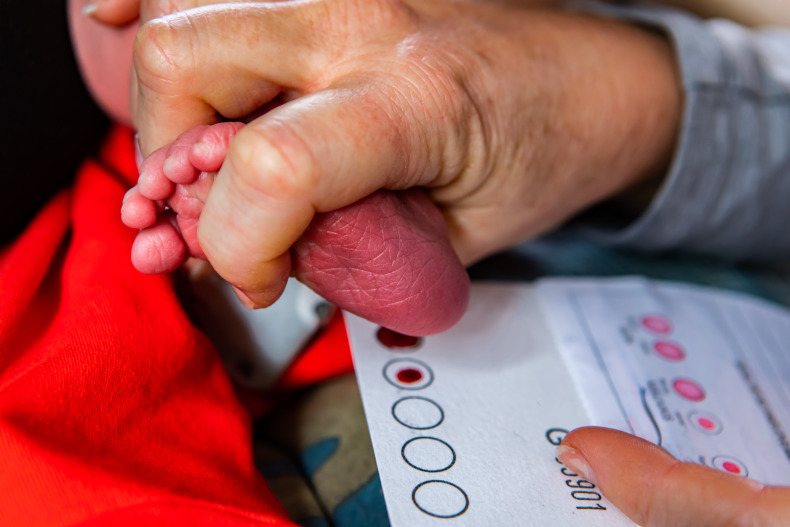
PHOTO: Dried blood spot sample collection in a newborn (Image source: Shutterstock)

In newborn screening, the stability of DBS samples facilitates the shipment of blood for specialised analysis offsite from the location of birth. DBS cards are typically prepared from small drops of blood, dried at room temperature, and can remain stable at room temperature for at least one week. These properties also make DBS-derived samples desirable for screening initiatives in LMICs. Collection and analysis of international DBS have provided an opportunity to test the feasibility of deriving population-level gestational age estimates and identifying and initiating treatment of participants with rare but treatable diseases [9].

The potential exists to use these samples for remote newborn screening and potentially other screening and diagnostic efforts. However, logistical challenges to this approach need to be examined. We describe the feasibility of international DBS sample collection for newborn screening analysis from the perspective of sample collection, shipping, and analysis turn-around times for samples collected in Bangladesh, Kenya, and Zambia and analysed in Ontario, Canada. We also consider the future opportunities for this approach.

## DBS COLLECTION, SHIPPING, AND ANALYSIS IN LOW-RESOURCE SETTINGS

We aimed to evaluate the use of a gestational age algorithm based on metabolic data from DBS samples collected in Kenya, Zambia, Zimbabwe, and Bangladesh. This project was a partnership between the Ontario Hospital Research Institute (OHRI), Newborn Screening Ontario (NSO), the Department of Pediatrics at the Stanford University School of Medicine, the Child Health Research Foundation in Mirzapur (Bangladesh), the Kenya Medical Research Institute (KEMRI) in Kisumu (Kenya), and the University of Zambia, Lusaka in collaboration with the University of North Carolina, Chapel Hill.

A detailed procedure manual was developed and staff at international partner sites were trained in person on the proper collection, storage, and shipping of cord and heel prick DBS samples. Pregnant women were enrolled during their first antenatal care visit or at childbirth. Cord and heel prick blood samples were collected from newborns and spotted on DBS cards, air dried at room temperature and stored up to a week before shipping to NSO. Upon receipt, the samples were visually assessed for quality and considered unsatisfactory if there was insufficient blood, serum rings, or if the sample was clotted, layered, or otherwise overtly damaged. A detailed study protocol can be found here [10].

NSO screens for over thirty priority diseases using immunoassays, enzyme assays, high performance chromatography, liquid chromatography-mass spectrometry (LC-MS/MS), Flow Injection Analysis Tandem Mass Spectrometry (FIA-MS/MS), and quantitative real time PCR (qPCR) [10,11]. Sample receipt and analysis were integrated into the existing NSO workflow. Further details on analytes measured can be found here [4,5,10]. Priority analysis times were put in place for all biochemical testing and hemoglobinopathy screening, with the goal of completing analysis of priority conditions within 14 days of birth.

Real-time notification of inborn metabolism errors was made for conditions that would benefit from immediate and accessible treatment at the partner sites. Metabolite integrity, potential for confirmatory testing, and availability of effective interventions were all taken into consideration in determining the feasible management of screen positive results, and our approach and rationale have been described in detail elsewhere [5,9,13]. Based on the above considerations, three high-priority conditions were identified for real-time reporting: congenital hypothyroidism, hemoglobinopathies, and medium-chain acyl-CoA dehydrogenase deficiency (MCADD).

Of the 4731 samples received, only three samples were completely excluded (0.04%) from analysis and 58 (1.2%) underwent partial analysis. The most common reason for incomplete analysis was insufficient quantity of blood to fill out the sample circles on the DBS card.

**Figure 1:**
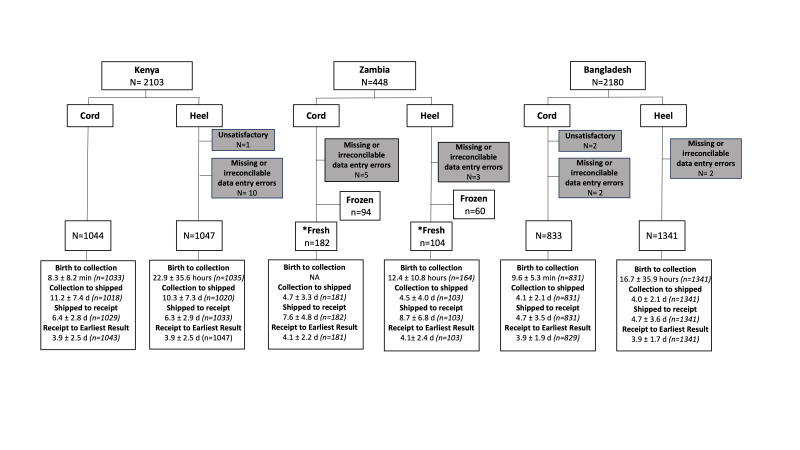
Summary of sample collection by site. 4731 samples were received at Newborn Screening Ontario (NSO) from Kenya, Zambia, and Bangladesh. Turn-around times for 1) birth to collection (sample collection date and time – birth date and time), 2) collection to shipped (shipping date and time – collection date and time), 3) shipped to receipt (date received at NSO – date shipped from site) and 4) receipt to earliest result (date and time of first results – date and time received at NSO). Some shipping and collection time data were unavailable. This is reflected in the sample sizes. *Data are only presented for fresh samples. At the Zambia site, n = 94 cord and n = 60 heel samples were collected, frozen and later shipped frozen (data not presented).

Cord samples were ideally collected within 30 minutes of delivery of the placenta and heel prick samples were collected 24-72 hours after birth or as late as possible, but before early hospital discharge. Most cord samples were collected within 30 minutes of birth, and most heel prick samples were collected before 24 hours due to hospital discharge occurring before then (**Figure 1**).

**Figure 2:**
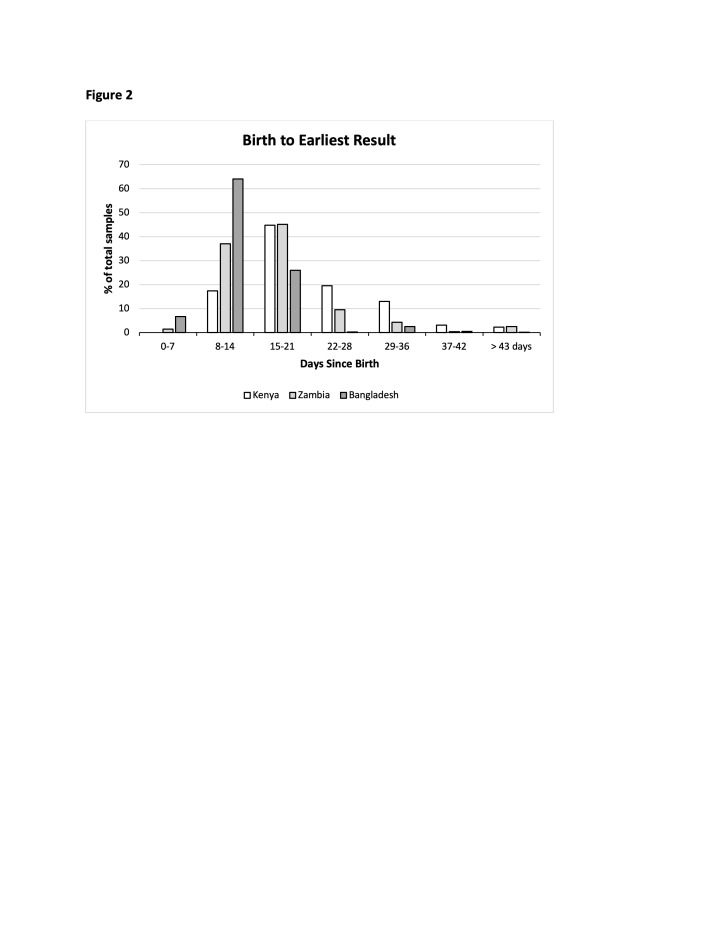
Distribution of birth to earliest result timing by site. Data are presented as percent of samples analysed per week since birth for cord and heel prick samples collected in Kenya, Bangladesh, and Zambia (fresh samples only).

On average, samples arrived within 6 days of shipping. Sample analysis timing at NSO varied depending on the testing method and disease. Diseases which can cause irreversible harm or death within the first weeks after birth use biochemical tests (ie, congenital hypothyroidism) to promptly identify the level of a substance in the sample and produce a screening result within 1-2 days. Samples were analysed for priority conditions within 4 days of receipt at NSO. **Figure 2** shows that most samples with priority analysis occurred within 3 weeks of birth.

## OUR OBSERVATIONS

Our experience suggests that remote population-based screening using shipped DBS samples under carefully controlled collection and shipping conditions is feasible. On average, samples arrived within 6 days of shipping and priority analysis occurred within 4 days of receipt at NSO or within 2 (Bangladesh) to 3 weeks (Kenya, Zambia) of birth (**Figure 2**). Thus, we were able to obtain both cord and heel prick samples and perform analysis at a centralised site on time. Sample quality was high, with only three samples being completely excluded from analysis. The current shipping and analysis procedures do not appear to affect pre-analytical quality or suitability for screening.

We have successfully implemented the workflow required to provide population-level estimates of gestational age in low resource settings [5]. Our analysis of collected samples and timing suggests that DBS samples could also be utilised for individual newborn screening or care guidance for LMICs when information is needed within the first few weeks of an infant’s life. Situations in which earlier assessments are needed may be more challenging. Our approach could be adapted for a shorter timeline if samples were shipped more frequently or shipped to a regional location, thus reducing shipping times.

## FUTURE OPPORTUNITIES

The adapted NSO workflow described here could be incorporated into demographic surveillance sites to support the scale-up of population-level estimates of gestational age, as well as more robust newborn screening programs, or screening of infants identified as at risk, such as preterm or SGA infants. Future research could also examine the regionalisation of such efforts, including reduced shipping distances to regional (rather than global) sites for analyte measurement.

This platform could also be expanded to screen for additional congenital metabolic and genetic disorders. We have already demonstrated the feasibility of international screening of hemoglobinopathies, MCADD, and congenital hypothyroidism [9], which were chosen since both confirmatory testing and treatment were accessible at study sites. Newborn screening could be expanded to identify other conditions, depending on the confirmatory testing, treatment capacity of the site, and birth-to-analysis turn-around times.

The use of DBS is particularly advantageous as sample collection is affordable, only requiring a lancet and blood spot card; furthermore, training for sample collection is minimal. DBS cards are ideal for shipping as they are relatively stable at room temperature for short-term storage [14], are affordable to ship, and are considered an exempt biological specimen, requiring no special shipping and handling. Long-term storage (-80°C) of DBS is much more cost-effective than of plasma, as DBS cards require very little storage space. Consideration could be given to adapting the DBS platform for other surveillance initiatives such as genetic testing, serological surveys (ie, population levels of COVID-19 antibodies), viral DNA testing (ie, HIV or cytomegalovirus prevalence or screening) or toxicological testing (ie, population-level testing for environmental toxins; drug use/exposure) [15]. Building an expanded set of screening initiatives on top of an existing program could further improve economies of scale.

With the current workflow, real-time reporting of gestational age estimates is not possible, due to the lag time required for molecular testing to occur (mean range of 32-43 days after birth in these cohorts) and generation of these estimates. Population estimates of gestational age can be obtained, however, and provide valuable information on preterm birth rates and inform global funding initiatives. Further research could evaluate the use of an algorithm based exclusively on biochemical test results, or prioritise key molecular testing markers (ie, TRECs) critical for gestational age estimates and thus allow for quicker gestational age reporting which could potentially inform infant care.

## CONCLUSION

We describe a viable program for the analysis of international DBS samples for gestational age estimates and newborn screening initiatives. This program could serve as a model for future international initiatives and could be further developed for other DBS testing initiatives. Future research should evaluate the possibility of real-time reporting of gestational age estimates back to the study site with modified algorithms. Further implementation of an expanded set of screen positive results back to international sites would also improve the cost-effectiveness of this programme.
